# A food-grade expression system for d-psicose 3-epimerase production in *Bacillus subtilis* using an alanine racemase-encoding selection marker

**DOI:** 10.1186/s40643-017-0139-7

**Published:** 2017-01-28

**Authors:** Jingqi Chen, Zhaoxia Jin, Yuanming Gai, Jibin Sun, Dawei Zhang

**Affiliations:** 1 Tianjin Institute of Industrial Biotechnology, Chinese Academy of Sciences, Tianjin, 300308 People’s Republic of China; 2 Key Laboratory of Systems Microbial Biotechnology, Chinese Academy of Sciences, Tianjin, 300308 People’s Republic of China; 3 School of Biological Engineering, Dalian Polytechnic University, Dalian, 116034 People’s Republic of China

**Keywords:** *Bacillus subtilis*, Cre/*lox* system, d-Psicose 3-epimerase, Fed-batch fermentation, Food-grade system

## Abstract

**Background:**

Food-grade expression systems require that the resultant strains should only contain materials from food-safe microorganisms, and no antibiotic resistance marker can be utilized. To develop a food-grade expression system for d-psicose 3-epimerase production, we use an alanine racemase-encoding gene as selection marker in *Bacillus subtilis*.

**Results:**

In this study, the d-alanine racemase-encoding gene *dal* was deleted from the chromosome of *B. subtilis* 1A751 using Cre/*lox* system to generate the food-grade host. Subsequently, the plasmid-coded selection marker *dal* was complemented in the food-grade host, and RDPE was thus successfully expressed in *dal* deletion strain without addition of d-alanine. The selection appeared highly stringent, and the plasmid was stably maintained during culturing. The highest RDPE activity in medium reached 46 U/ml at 72 h which was comparable to RDPE production in kanamycin-based system. Finally, the capacity of the food-grade *B. subtilis* 1A751D2R was evaluated in a 7.5 l fermentor with a fed-batch fermentation.

**Conclusion:**

The alanine racemase-encoding gene can be used as a selection marker, and the food-grade expression system was suitable for heterologous proteins production in *B. subtilis*.

**Electronic supplementary material:**

The online version of this article (doi:10.1186/s40643-017-0139-7) contains supplementary material, which is available to authorized users.

## Background

d-Psicose is a hexoketose monosaccharide sweetener, which is a C-3 epimer of d-fructose and is rarely found in nature (Mu et al. 2012). It has 70% relative sweetness but 0.3% energy of sucrose and is suggested as an ideal sucrose substitute for food products (Matsuo et al. 2002; Oshima et al. 2006). It shows important physiological functions, such as blood glucose suppressive effect (Hayashi et al. 2010; Iida et al. 2008), reactive oxygen species scavenging activity (Matsuo et al. 2003), and neuroprotective effect (Takata et al. 2005). It also improves the gelling behavior and products good flavor during food process (Sun et al. 2004). In virtue of its outstanding advantages, the conversion of d-fructose to d-psicose using the d-psicose 3-epimerase has been investigated for the commercial production of d-psicose.

*Bacillus subtilis* is a food-safe microorganism, which has been used to food fermentation for a long period of time (Song et al. 2015). Several gene expression systems have been developed for high-level production of heterologous proteins such as amylase (Chen et al. 2015a, b), lysozyme (Zhang et al. 2014), protease (Degering et al. 2010), and lipase (Lu et al. 2010). However, few reports are concerned with application of recombinant *B. subtilis* directly to food processing. One important reason is that most vectors (mainly based on antibiotics) used in this systems are not food-grade. Food-grade expression systems have been widely developed and investigated for lactic acid bacteria. Such systems require that the resultant strains should only contain materials from food-safe microorganisms, and no antibiotic resistance marker can be utilized. Usually, food-grade selection markers can be classified as dominant markers or complementation markers (de Vos 1999). Compared with dominant markers, selection markers based on complementation do not require supplements in the cultivation medium. In order to develop a food-grade complementation-based system, usually a gene on the host chromosome is mutated or deleted, and a wild type copy is inserted into the expression vector. The alanine racemase gene *dal* is involved in the conversion of d-alanine and l-alanine (Bron et al. 2002), and d-alanine is not a common ingredient of large-scale fermentation media (Nguyen et al. 2011); the *dal* gene thus has considerable potential as a food-grade selection marker in *B. subtilis*.

In the present study, we developed a food-grade expression system for the production of d-psicose 3-epimerase (RDPE) from *Ruminococcus* sp. 5_1_39BFAA in *B. subtilis*, using alanine racemase gene *dal* as the selection marker. The selection appeared highly stringent, and the plasmid was stably maintained during culturing. Moreover, the expression level of RDPE in the newly developed food-grade system was comparable to the level obtained in the conventional kanamycin-based system. This new expression system was therefore suitable for food-grade production of various heterologous proteins.

## Methods

### Bacterial strains, plasmids, and growth conditions

Bacterial strains and plasmids used in this study are listed in Additional file 1: Table S1. *Escherichia coli* DH5α was used as a host for cloning and plasmid preparation. *Bacillus subtilis* 1A751, which is deficient in two extracellular proteases (*nprE, aprE*), served as the parental strain. The plasmid pMA5 is an *E. coli/B. subtilis* shuttle vector and used to clone and express protein. The plasmids p7Z6 containing *lox*71-*zeo*-*lox*66 cassette and p148-cre containing *cre* expression cassette were used for the knockout of target gene. Transformants of *E. coli* and *B. subtilis* were selected on Luria–Bertani (LB) agar [1% (w/v) peptone, 0.5% (w/v) yeast extract, 1% (w/v) NaCl, and 2% (w/v) agar], supplemented with ampicillin (100 μg/ml), zeocin (20 μg/ml), or kanamycin (50 μg/ml) depending on the plasmid antibiotic marker. *E. coli* DH5α was incubated in LB medium supplemented with ampicillin (100 μg/ml) at 37 °C. *Bacillus subtilis* was cultivated in SR medium [1.5% (w/v) peptone, 2.5% (w/v) yeast extract, and 0.3% (w/v) K_2_HPO_4_, pH 7.2] containing additionally kanamycin (50 μg/ml) or zeocin (20 μg/ml) at 37 °C. All of the strains were incubated under a shaking condition at 200 rpm. Except the fed-batch fermentation, all of the experiments were repeated at least 3 times, and mean values were used for comparison.

### Primers and oligonucleotides

Polymerase chain reaction (PCR) primers and oligonucleotides used in this study were synthesized by GENEWIZ (Suzhou, China) and listed in Additional file 1: Table S2.

### Genetic manipulation

PCRs were performed using PrimeSTAR Max DNA Polymerase (TaKaRa, Japan). DNA fragments and PCR products were excised from a 0.8% agarose gel and purified by E.Z.N.A.™ Gel Extraction Kit (200) (Omega Bio-tek, Inc., USA) according to the manufactures’ instruction. E.Z.N.A.™ Plasmid Mini Kit I (Omega Bio-tek, Inc., USA) was applied for plasmid extraction according to the manufactures’ instruction. Genomic DNA isolation was carried out by TIANamp Bacteria DNA Kit (TIANGEN BIOTECH (BEIJING) CO., LTD., China). All the DNA constructs were sequenced by GENEWIZ (Suzhou, China).

### Construction of the *dal* deletion mutant

The deletion of the alanine racemase gene *dal* in *B. subtilis* was performed using Cre/*lox* system as described previously (Yan et al. 2008; Dong and Zhang 2014). The two flanking fragments upstream and downstream (~1 kb) of the *dal* gene were amplified using genomic DNA from *B. subtilis* 168 as template and UP-F/UP-R and DN-F/DN-R as primers, respectively. The *lox*71-*zeo*-*lox*66 cassette (~0.5 kb) was amplified from the plasmid p7Z6 using the primers lox-F and lox-R. Then, the flanking fragments and the *lox*71-*zeo*-*lox*66 fragment were fused together by splicing by overlap extension PCR (SOE-PCR) using the primers UP-F and DN-R. Subsequently, the fused fragment was transformed into *B. subtilis* 1A751. Selection of the double crossover mutant (*B. subtilis* 1A751D1), marker excision of by Cre-dependent recombination of the *lox*-sites, and selection of the *dal* deletion mutant (*B. subtilis* 1A751D2) were performed by the previous strategy (Yan et al. 2008). Finally, the entire *dal* gene was thus successfully deleted via double crossing over and marker excision in the chromosome of *B. subtilis* 1A751, which was further confirmed by PCR amplification.

### Construction of plasmids

The food-grade expression plasmid was constructed based on pMA5 by replacing the zeocin resistance gene *zeo* with the alanine racemase gene *dal* from *B. subtilis* 168 using a sequence-independent method named “simple cloning” developed by Chun You (2012). Based on the nucleotide sequence of *dal*, the primers dal-F/dal-R were designed to amplify the fragment *dal* using the *B. subtilis* 168 as the template. The linear vector backbone was amplified using the primers pMA5-F1 and pMA5-R1 as the primers and the plasmid pMA5 as the template. Dal-F/dal-R had the reverse complementary sequences of pMA5-F1/pMA5-R1, respectively. Then, the DNA multimer was generated based on these DNA templates by prolonged overlap extension PCR (POE-PCR). Eventually, the POE-PCR products (DNA multimer) were directly transformed into competent *E. coli* DH5α, yielding the recombinant plasmid pMA5-DAL. Likewise, the *rdpe* gene from pET-RDPE was inserted into the plasmid pMA5-DAL downstream of the promoter P_*Hpa*II_, resulting into the recombinant plasmid pMA5-DAL-RDPE.

### Stability of the recombinant plasmids

The evaluation of the stability of the plasmid pMA5-DAL-RDPE was conducted using the method described by Nguyen (2005). The recombinant strains were inoculated onto Plate A (LB agar plate without supplement of d-alanine with selection pressure) and Plate B (LB agar plate with supplement of 200 μg d-alanine/ml without selection pressure). Colony numbers of the strain 1A751D2R on Plate A and Plate B were named as *C*_A_ and *C*_B_. The value of *C*_A_/*C*_B_ was regarded as the stability of the plasmid pMA5-DAL-RDPE at the certain generation of cultivation.

### Fed-batch fermentation in 7.5 l fermentor

The food-grade RDPE production in *B. subtilis* 1A751D2R was evaluated in 7.5 l BIO FLO 310 fermentor (New Brunswick Scientific co Inc., USA) with a fed-batch strategy. The airflow rate was 6.0 l/min, and dissolved oxygen tension was maintained between 20 and 40% air saturation by automatic adjustment of speed of the stirrer. The temperature was kept at 37 °C and the pH was controlled at pH 7.2. Foam was controlled by the addition of a silicone-based anti-foaming agent. The fermentation medium was SR medium. The fermentation was performed with an initial working volume of 3.5 l. When the cell growth rate became constant, the substrate fed-batch mode was started by adding 8.0% soluble starch at a constant flow rate, until the final concentration of soluble starch was up to 4.0%. Cell growth was monitored by measuring dry cell weight of the fermentation broth. The activity of RDPE was determined by measuring the supernatant of broth.

### Enzyme assays

The RDPE activity was analyzed by determining the amount of d-psicose obtained from d-fructose. One milliliter of reactions mixture contained d-fructose (20 g/l) in sodium phosphate buffer (50 mM, pH 8.0) and 200 μl crude enzyme. The reaction was incubated at 55 °C for 10 min,following by boiling at 100 °C for 10 min. The obtained d-psicose in the mixture was determined via high-performance HPLC system with a refractive index detector and a Sugar-PakTM column (6.5 mm × 300 mm; Waters), which was eluted with ultrapure water at 80 °C and 0.4 ml/min. One unit of DPEase activity is defined as the amount of enzyme that catalyzed the production of 1 μmol d-psicose per minute. For the determination of extracellular enzyme activity, the crude enzyme was the supernatant of fermentation broth. For the determination of intracellular enzyme activity, the cells need to be broken. *Bacillus subtilis* cells expressing RDPE were harvested from the culture broth by centrifugation at 6000×*g* for 10 min at 4 °C. The cells were then suspended in lysis buffer (25 mM Tris/HCl, 300 mM NaCl, and 40 mM imidazole, pH 8.0). The suspended cells were disrupted using a high-pressure homogenizer (APV, Denmark) at 900–1000 bar. The supernatant was obtained by centrifugation at 15,000×*g* for 30 min at 4 °C and filtration through a 0.45 μm filter. Then, the crude extract was applied in the enzyme assay.

### SDS-PAGE analysis

Culture samples (1 ml) were harvested and the supernatant was separated from the culture medium by centrifugation (12,000*g*, 10 min, 4 °C). After adding 5× SDS-PAGE sample buffer, the supernatants were boiled for 10 min, and proteins were separated in SDS-PAGE using the NuPAGE 10% Bis–Tris Gel (Novex by Life Technologies, USA) in combination with MOPS SDS Running Buffer (Invitrogen Life Technologies, USA). PageRuler Prestained Protein Ladder (Invitrogen Life Technologies, USA) was used to determine the apparent molecular weight of separated proteins. Proteins were visualized with Coomassie Brilliant Blue.

## Results and discussion

### Construction of the food-grade host strain with deficiency of *dal*

**Fig. 1:**
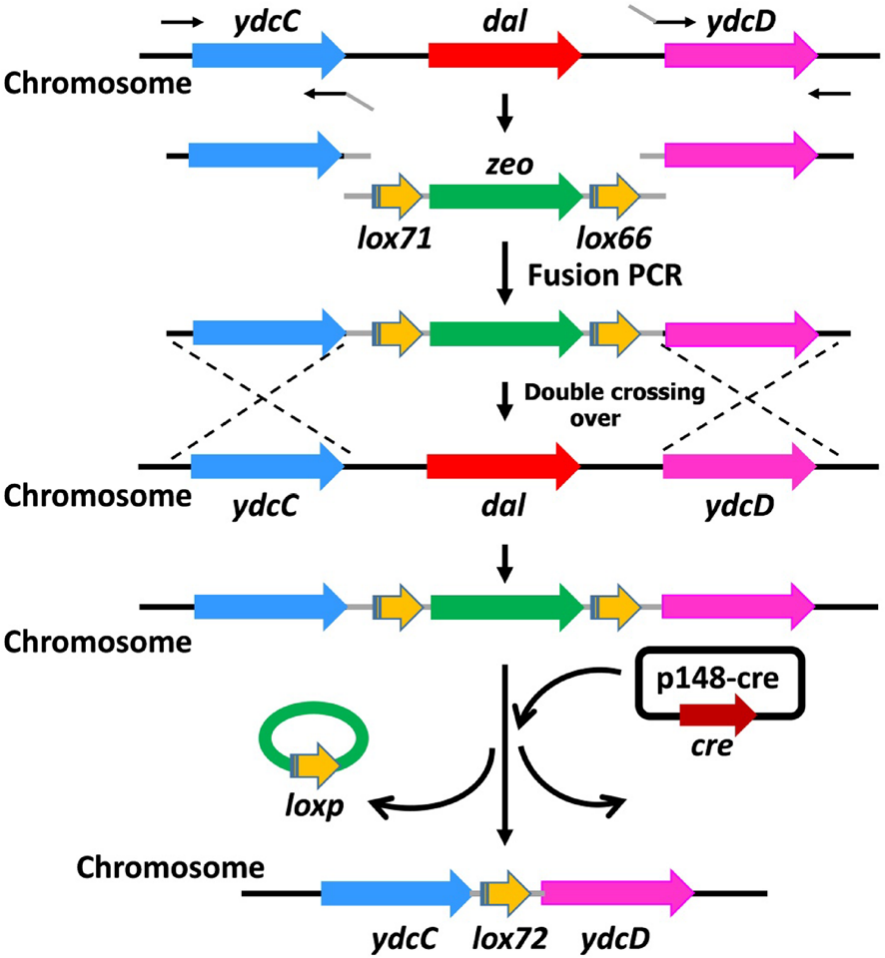
Schedule for the deletion of *dal* using Cre/lox system: (1) the front and back regions flanking the target gene to be deleted were PCR amplified, gel purified, and fused by PCR. The fragment *lox*71-*zeo*-*lox*66 was cloned from the plasmid p7Z6. (2) PCR-fused products were directly used to transform *B. subtilis*, and Zeo^r^ transformants were selected. (3) p148-cre was introduced into a Zeo^r^ clone, and the recombination between lox71 and lox66 was mediated by expressed Cre recombinase. (4) p148-cre was eliminated to get the target strain by 5 times sub-cultivation

In order to obtain the mutant strain with deficiency of *dal*, we attempted to knock out the gene *dal* from *B. subtilis* chromosome using Cre/*lox* system. The flow chart for construction of the food-grade host strain is shown in Fig. 1. The two flanking fragments upstream and downstream of *dal* and the fragment *lox*71-*zeo*-*lox*66 cassette were fused into a long DNA fragment by SOE-PCR, and the fused fragment was then directly transformed into *B. subtilis* 1A751. Because the fused fragment had two efficient homology regions with the chromosome of *B. subtilis* 1A751, the homologous recombination via double crossing over event thus occurred, and the *lox*71-*zeo*-*lox*66 cassette was integrated into the chromosome. The transformants (zeocin-resistant phenotype) were selected on LB agar plate with the addition of 20 μg zeocin/ml and 200 μg d-alanine/ml. To exclude the false positive, the transformants with zeocin-resistant phenotype were replica-plated on LB agar plate and LB agar plate with the supplement of zeocin and d-alanine. The strains with the *lox*71-*zeo*-*lox*66 cassette could not grow on LB agar plate but grown on LB agar plate with the supplement of zeocin and d-alanine. Of 36 tested transformants, 28 displayed the desired phenotype (Additional file 1: Figure S1a); 5 of these were confirmed by PCR, cultivated in liquid LB medium (Additional file 1: Figure S1b) and named as *B. subtilis* 1A751D1. The plasmid p148-cre with P_*spac*_-*cre* cassette was transformed into 1A751D1 and selected on LB agar plate with kanamycin and d-alanine. With the induction of IPTG, the Cre recombinase was expressed and the *lox*-sequence-flanked zeocin resistance gene was then excised. 30 colonies were identified by PCR and 100% of these lost *zeo* gene, indicating high efficiency of gene deletion using Cre/lox system. The obtained strain 1A751D1C was subcultured in liquid LB medium with d-alanine five times to lose the plasmid p148-cre and then incubated on LB agar plate with d-alanine, followed by replica plating on LB agar plate with d-alanine and LB agar plate with kanamycin and d-alanine. 23 of 25 tested colonies displayed the desired phenotype; 5 of these were further identified by PCR, with loss of the plasmid p148-cre because of the plasmid instability. At last, we successfully obtained the d-alanine-auxotrophic strain 1A751D2.

### Construction of food-grade expression plasmids with auxotrophic marker

**Fig. 2:**
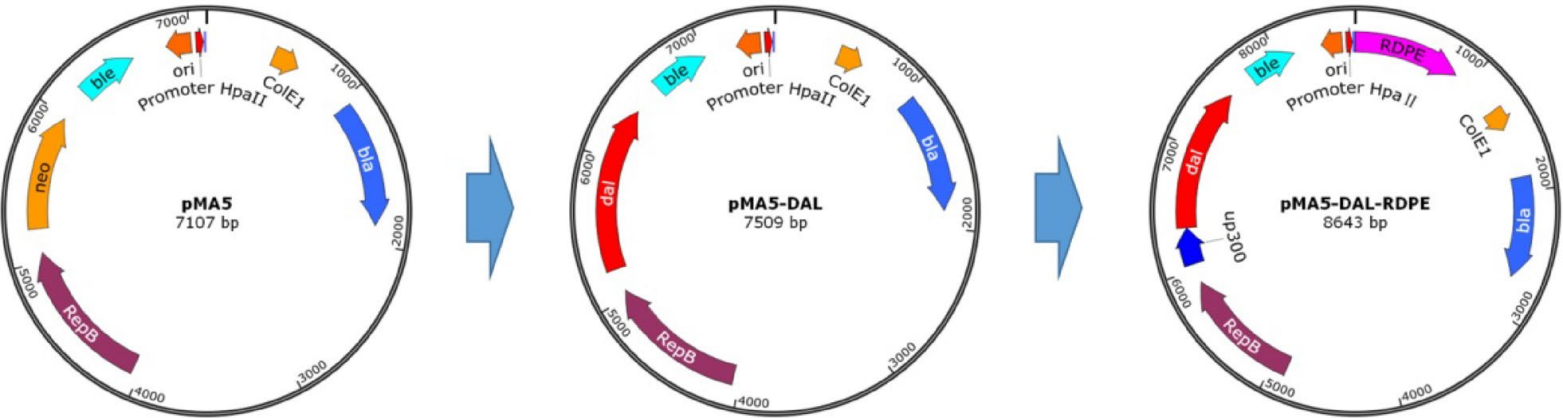
Construction of the food-grade expression plasmid pMA5-DAL-RDPE

As described in “Methods” section and shown in Fig. 2, the new expression plasmids were constructed based on the conventional plasmid pMA5. First, the *neo* gene of pMA5 was replaced with the gene *dal*, and *dal* was under the control of the native promoter of the *neo* gene, yielding the plasmid pMA5-DAL. Then, the gene *rdpe* encoding d-psicose 3-epimerase was cloned and inserted into pMA5-DAL downstream of a strong and constitutive promoter P_*Hpa*II_ which is from *Staphylococcus aureus*, resulting into the food-grade expression plasmid pMA5-DAL-RDPE.

### Expression of d-psicose 3-epimerase in the food-grade system

**Fig. 3:**
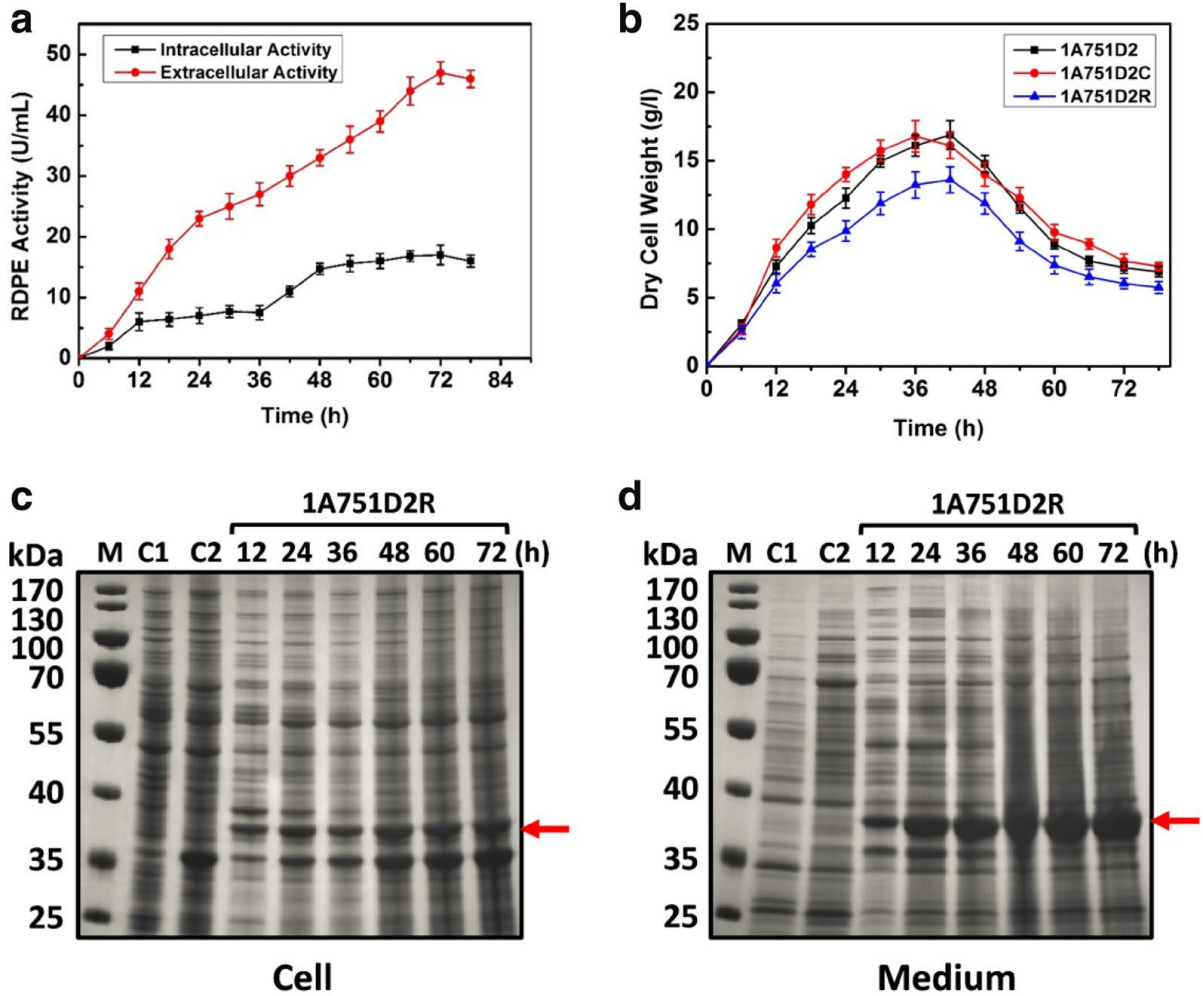
Expression of RDPE in the food-grade system: **a** the intra- and extracellular activity analysis of RDPE in 1A751D2R. **b** The growth of the recombinant strains. **c** The SDS-PAGE analysis of intracellular RDPE in 1A751D2R; *C1* the sample of 1A751D2 at 72 h, *C2* the sample of 1A751D2C at 72 h. **d** The SDS-PAGE analysis of extracellular RDPE in 1A751D2R; *C1* the sample of 1A751D2 at 72 h, *C2* the sample of 1A751D2C at 72 h

The food-grade plasmids pMA5-DAL-RDPE and pMA5-DAL were transformed into the food-grade host strain 1A751D2 with the deficiency of alanine racemase gene to generate the recombinant strain 1A751D2R and 1A751D2C, respectively. Subsequently, the strains 1A751D2R, and 1A751D2C was inoculated in 250 ml shake flask containing 30 ml SR medium at 37 °C and 200 rpm for 78 h. The strain 1A751D2 was inoculated in SR medium with the addition of 200 μg d-alanine/ml as the negative control. The activity of RDPE in the cells or medium was determined throughout all the cultivation process. As shown in Fig. 3a, the extracellular activity of RDPE was gradually increased with the fermentation process, and the highest activity reached 46 U/ml at 72 h, which was comparable to RDPE production in the conventional *neo*-based system (44 U/ml at 72 h). In our previous study, we have demonstrated that RDPE is one of non-classically secreted proteins which are secreted via non-classical secretion pathway in *B. subtilis* (Chen et al. 2016). Thus, RDPE can still be exported into the extracellular milieu without any classical signal peptide. To provide further evidence supporting the above result, the SDS-PAGE analysis was performed. Distinct bands with a molecular mass of about 34 kDa were observed which was in good agreement with the deduced value (Fig. 3d), and the result was consistent with the activity analysis. Meanwhile, we noted that there was still residual RDPE in the cell fraction over the whole fermentation (Fig. 3a, c). We also traced the growth states of the recombinant strains. As shown in Fig. 3b, there was no difference between the biomass of 1A751D2 and that of 1A751D2C, however, the biomass of 1A751D2R was slightly lower than that of 1A751D2 and 1A751D2C. This was caused by increased metabolic burden with the overexpression of RDPE, because high protein production was usually accompanied by reduced growth rates. In addition, the biomass of all the three strains sharply decreases after about 40 h, we speculated that the carbon source might have been depleted at that time.

### Evaluation of plasmid stability and copy numbers

The stability of the *dal*- and *neo*-based plasmids was determined using pMA5-DAL-RDPE (*dal*) in *B. subtilis* 1A751D2 and pMA5-RDPE (*neo*) in *B. subtilis* 1A751. The strains were cultivated for an estimated 80 generations (160 h) at 37 °C in selective and nonselective medium, followed by replica plating of diluted cultures in order to determine the stability of plasmids. The fraction of cells retaining the plasmid pMA5-RDPE after 80 generations in nonselective medium (SR without kanamycin) was 0%, whereas in selective medium 85% of the colonies still contained the plasmid. Interestingly, the plasmid pMA5-DAL-RDPE showed much better stability: after 80 generations, it was retained in about 30 and 100% of cells of *B. subtilis* 1A751D2R under nonselective and selective conditions, respectively (Table 1).

**Table 1:** Stability of *dal*-based and *neo*-based plasmids in different medium

Plasmid	5 Generations (%)	15 Generations (%)	40 Generations (%)	80 Generations (%)
pMA5-RDPE/SR	56	10	3	0
pMA5-RDPE/SR + Kan	100	95	88	85
pMA5-DAL-RDPE/SR	100	100	100	100
pMA5-DAL-RDPE/SR + d-alanine	88	76	54	30

*Bacillus subtilis* 1A751R harboring pMA5-RDPE and *B. subtilis* 1A751D2R harboring pMA5-DAL-RDPE were cultivated in selective and nonselective medium. The kanamycin concentration was 50 μg/ml; the d-alanine concentration was 200 μg/ml. The strains were cultivated at 37 °C

Every 10 h, 5 generations passed. The stability of plasmids was calculated by dividing the number of colonies on selective medium with the number of colonies on nonselective medium

### Food-grade production of RDPE in 7.5 l fermentor with fed-batch fermentation

**Fig. 4:**
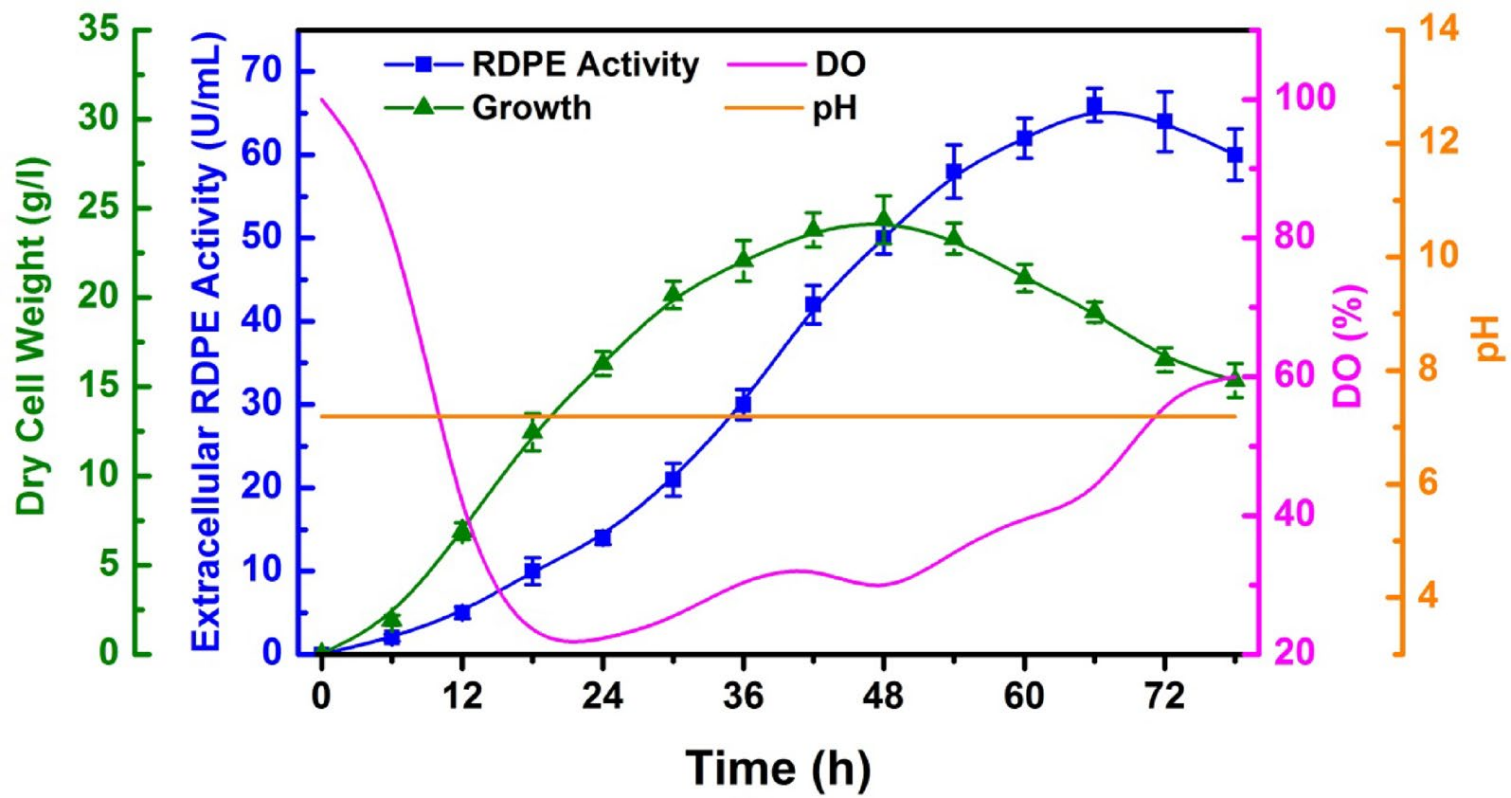
Production of RDPE in recombinant strain 1A751D2R by fed-batch fermentation in 7.5 l fermentor. *Blue line* RDPE activity in medium. *Green line* biomass. *Pink line* DO concentration. *Orange line* pH

The expression efficiency of *B. subtilis* 1A751D2R was further explored in 7.5 l fermentor. The fermentor was inoculated with 5% (v/v) of freshly cultured 1A751D2R grown in SR medium at 37 °C for 18 h. To maintain cell growth and RDPE production, we chose a fed-batch strategy. When the cell growth rate was constant, 8.0% (w/v) soluble starch was added at a constant flow rate until the final concentration of soluble starch was up to 4.0% (w/v). As shown in Fig. 4, during the growth phase, the maximum biomass in the fermentor reached 23.9 g/l (dry cell weight) at 48 h. Compared with the biomass decrease in shake flask, the biomass decrease after 48 h in 7.5 l fermentor was very mild, which was attributed to the supplement of soluble starch. The activity of RDPE in medium was continuously increased and reached the maximum of 65 U/ml with a high productivity of 0.9 U/ml h at 72 h. The RDPE concentration in the supernatant reached about 1.8 g/l. The high activity of RDPE indicates that *B. subtilis* is a suitable host for the industrial production of heterologous protein.

## Conclusion

In this study, we developed a food-grade expression system for d-psicose 3-epimerase production in *B. subtilis*. The plasmid co-expressing *rdpe* and *dal* was introduced into *dal* mutant, selection appeared highly stringent, and plasmids were stably maintained during culturing. Moreover, the production of RDPE in this food-grade expression system was comparable to that in *neo*-based system. The results showed that this system was very suitable for food-grade expression of heterologous proteins.

## Additional file

**Additional file 1: Figure S1**. The isolation of *B. subtilis* 1A751D1. **Table S1.** Strains and plasmids used in this study. **Table S2.** Primers used in this study.

## Abbreviations

RDPE: d-psicose 3-epimerase from *Ruminococcus* sp. 5_1_39BFAA
*Dal*: d-alanine racemase-encoding gene
LB: Luria–Bertani
SR: super rich
PCR: polymerase chain reaction
SOE-PCR: splicing by overlap extension PCR
POE –PCR: prolonged overlap extension PCR
HPLC: high-performance liquid chromatography
SDS-PAGE: sodium dodecyl sulfate polyacrylamide gel electrophoresis
IPTG: isopropyl-β-d-thiogalactoside
g: grams
l: liter
h: hours
DO: dissolved oxygen
DCW: dry cell weight
