# Supplementary material for: A food-grade expression system for d-psicose 3-epimerase production in Bacillus subtilis using an alanine racemase-encoding selection marker
Source: Bioresour Bioprocess. 2017 Jan 28;4(1):9. doi: 10.1186/s40643-017-0139-7 (PMC5274643; doi:10.1186/s40643-017-0139-7)
Supplement: Supplementary file 1 — Additional file 1: Figure S1. The isolation of B. subtilis 1A751D1. Table S1. Strains and plasmids used in this study. Table S2. Primers used in this study. [file 40643_2017_139_MOESM1_ESM.docx]

**Supplementary figure**

**
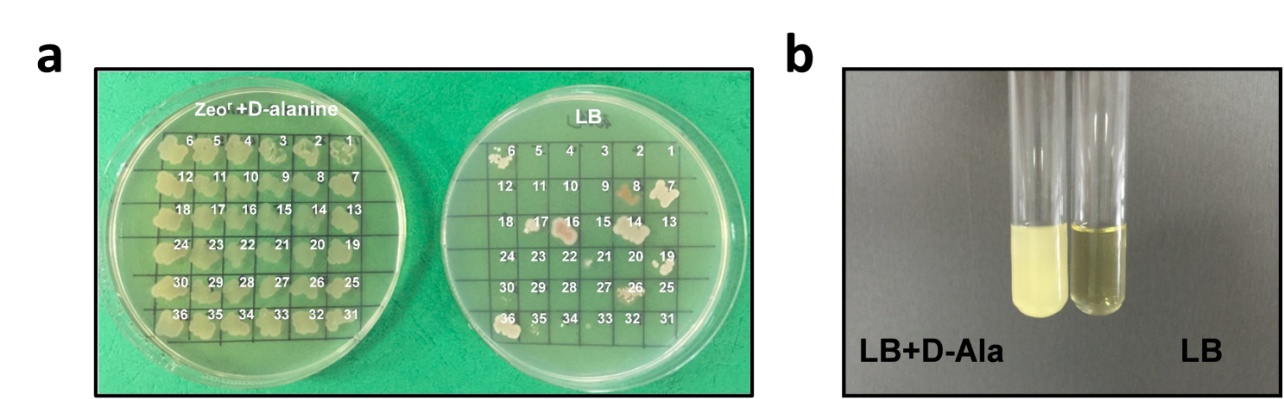
**

**Figure S1** The isolation of *B. subtilis* 1A751D1. **a** The transformants with zeocin-resistant phenotype was replica-plated on LB agar plate and LB agar plate with zeocin and D-alanine. **b** *B. subtilis* 1A751D1 was inoculated into liquid LB medium and liquid LB medium with zeocin and D-alanine.

**Table S1**

Strains and plasmids used in this study

| **Strains** | **Genotype and/or relevant characteristic(s)** | **Source** |
| --- | --- | --- |
| *E. coli* DH5α | F^-^∆*lac*U169(Ø80d *lac*Z∆M15) *sup*E44 *hsd*R17 *rec*A1 *gyr*A96 *end*A1 *thi*-1 *rel*A1 | Invitrogen |
| *B. subtilis* 168 | *trpC2* | Lab stock |
| *B. subtilis* 1A751 | *egl*S∆102 *bgl*T/*bgl*S∆EV *apr*E *npr*E *his* | BGSC^a^ |
| 1A751R | 1A751 containing pMA5-RDPE; Km^r^ | Lab stock |
| 1A751D1 | 1A751 with integration of *lox*71-*zeo*-*lox*66 (*dal*:: *lox*71-*zeo*-*lox*66); Zeo^r^ | This work |
| 1A751D1C | 1A751D1 containing p148-cre; Km^r^ | This work |
| 1A751D2 | 1A751 with integration of *loxP* (*dal*::*loxP*) | This work |
| 1A751D2C | 1A751D2 containing pMA5-DAL; Km^r^ | This work |
| 1A751D2R | 1A751D2 containing pMA5-DAL -RDPE; Km^r^ | This work |
| **Plasmids** |  |  |
| pET-RDPE | pET-28a derivative, *rdpe*; Ap^r^ | Lab stock |
| pMA5 | *E. coli/B. subtilis* shuttle vector, P*_HpaⅡ_*; Ap^r^, Km^r^ | Lab stock |
| pMA5-RDPE | pMA5 derivative, *rdpe* | Lab stock |
| p7Z6 | pMD18-T containing *lox*71-*zeo*-*lox*66 cassette | Lab stock |
| p148-cre | Ap^r^, Km^r^, P*_spac_*-*cre* expression cassette | Lab stock |
| pMA5-DAL | pMA5 derivative, *neo*::*dal*, Zeo^r^ | This work |
| pMA5-DAL-RDPE | pMA5-DAL derivative, *rdpe* | This work |

^a^ BGSC, *Bacillus* Genetic Stock Center, USA

**Table S2**

Primers used in this study

| Prmier | Sequence(5’-3’) |
| --- | --- |
| UP-F | CTGGCACAAAAAGCCGTCTCTGTAC |
| UP-R | TACGACACTTCCTAGCTTTTATTCAATATC |
| DN-F | TAACTTACCTAAATGGAGAATTCATAAAACAG |
| DN-R | CTTTTAGTGTGTCAGTCCACAGTTGG |
| lox-F | GATATTGAATAAAAGCTAGGAAGTGTCGTATACCGTTCGTATAGCAT |
| lox-R | CTGTTTTATGAATTCTCCATTTAGGTAAGTTATACCGTTCGTATAATGT |
| YZ-F1 | ACCATCGAGACAGGGAGTGAGC |
| YZ-F2 | TTTCATACACTTGGTCAGTCAGCTG |
| pMA5-F1 | CCTTTATTGCAGGTAAATATAAGCAATTAATGAACGATGACCTCTAATAATTGTTAATC |
| pMA5-R1 | CTCTGTAAAAAGGTTTTGTGCTCATTACGACACTTCCTAGCTTTTATTCA |
| dal-F | TGAATAAAAGCTAGGAAGTGTCGTAATGAGCACAAAACCTTTTTACAGAG |
| dal-R | GATTAACAATTATTAGAGGTCATCGTTCATTAATTGCTTATATTTACCTGCAATAAAGG |
| YZ-F2 | AGCTGAATAAGAACGGTGCTCTCC |
| YZ-R2 | CCTTTATTCCGTTAATGCGCCATG |
| pMA5-F2 | GTAAAACATGTATTTGAAGTCTAAGGATCCTCTAGAGTCGAGCTCAAG |
| pMA5-R2 | AAGCGTAATAAATACCATATTTCATATGTAAATCGCTCCTTTTTAGGT |
| RDPE-F | ACCTAAAAAGGAGCGATTTACATATGAAATATGGTATTTATTACGCTT |
| RDPE-R | CTTGAGCTCGACTCTAGAGGATCCTTAGACTTCAAATACATGTTTTAC |
| YZ-F3 | AACGGACAAAATAAAAATTGGCAAG |
| YZ-R3 | ATTGCGAATAATAATTTTTTCACGTTG |
